# Bacteriophage-Based Therapeutic, Diagnostic, and Biocontrol Platforms: Engineering, Evidence, and Translational Challenges

**DOI:** 10.3390/ijms27156615

**Published:** 2026-07-24

**Authors:** Nan Chen, Yingli Yang, Yao Wang, Yao Yao, Caihong Zheng

**Affiliations:** Department of Pharmacy, Women’s Hospital School of Medicine, Zhejiang University, Hangzhou 310006, China; chennan2719@zju.edu.cn (N.C.); yangyingli0110@163.com (Y.Y.); jidantou@outlook.com (Y.W.)

**Keywords:** bacteriophages, phage therapy, phage diagnostics, biocontrol, phage persistence, antimicrobial resistance, translational challenges

## Abstract

Bacteriophages are being revisited as programmable platforms for therapy, diagnostics, and biocontrol. Their value, however, depends on the setting. Therapeutic phages must do more than lyse bacteria in vitro: they need to reach infection sites, persist long enough to act, reduce bacterial burden, and limit resistance under clinically relevant conditions. Diagnostic platforms are judged by another standard, including sensitivity, specificity, matrix tolerance, and stable signal readout. Food, agricultural, and environmental applications instead rely on formulation stability, host specificity, scalable delivery, and ecological safety. This review summarizes the biological and engineering principles that support phage-based platforms, and then evaluates therapeutic, diagnostic, and nonclinical uses through an application-specific evidence framework. For therapy, we focus on evidence hierarchy, active phage exposure, immune clearance, persistence, infection spread, and host-resistance-bypass phenotypes. Recent studies on high-persistence and hyper-aggressive phages suggest that dissemination, plaque expansion, and resistance-bypass behavior should be considered during early candidate selection. Phage cocktails, antibiotic combinations, and engineered phages remain useful, but they should be treated as adaptive strategies rather than universal solutions. Overall, phage technologies require validation frameworks that link biological function with manufacturing quality, regulatory feasibility, and meaningful clinical or environmental endpoints.

## 1. Introduction

The global burden of antimicrobial resistance (AMR) continues to intensify, creating demand for antibacterial strategies that can complement or extend conventional antibiotics [1]. Bacteriophages are being reconsidered because they combine bacterial specificity, self-amplifying infection dynamics, genetic programmability, and compatibility with therapeutic, diagnostic, biocontrol, and microbiome-oriented workflows.

In clinical therapy, phages are increasingly studied as adjunctive or personalized agents for multidrug-resistant infections. Representative randomized and compassionate-use studies support feasibility and selected clinical promise, but the evidence remains heterogeneous and should not be interpreted as proof of routine phage monotherapy [2,3]. A clear distinction is therefore needed between in vitro activity, compassionate-use experience, and controlled clinical efficacy.

Phage-derived diagnostic platforms exploit receptor-binding proteins, reporter phages, and phage-templated amplification to support pathogen detection and point-of-care testing [4,5]. Outside clinical medicine, practical phage applications are most established in food biopreservation and plant protection, while fermentation control, wastewater biofilm management, and microbial-ecology interventions remain important emerging contexts [6,7].

These applications share a phage-based foundation but require different standards of evidence. This review therefore uses selective, application-specific coverage rather than an encyclopedic catalog. We distinguish engineering feasibility from platform performance and real-world utility. The analysis focuses on clinical evidence, active phage exposure, resistance evolution, diagnostic matrix performance, manufacturing quality, regulatory translation, and ecological safety. Table 1 illustrates these differences through representative approved, marketed, and investigational phage products across food safety, agricultural or veterinary use, and human therapy.

**Figure 1:**
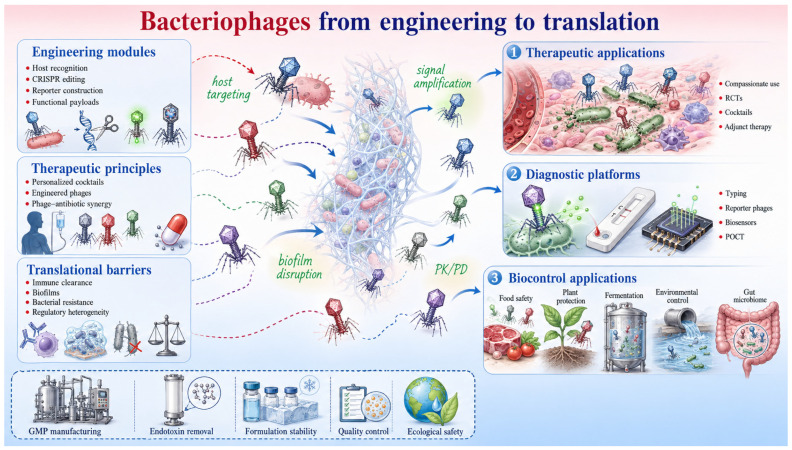
Bacteriophages from engineering to translation. The schematic organizes phage technologies into engineering modules, application domains, and translational barriers. Engineering strategies include host-recognition optimization, CRISPR-assisted genome editing, reporter construction, and functional payload delivery. These modules support therapeutic phage use, diagnostic platforms, and nonclinical biocontrol in food, agriculture, fermentation, environmental management, and microbiome modulation. Key barriers include immune clearance, resistance evolution, biofilm penetration, GMP-compliant manufacturing, endotoxin removal, formulation stability, standardized quality control, regulatory heterogeneity, and ecological safety.

The product examples in Table 1 show how uneven the field is in regulatory and clinical maturity. Figure 1 then places these examples within a broader framework, linking phage engineering modules to therapeutic, diagnostic, and biocontrol uses, as well as to the validation steps required before translation.

## 2. Biology of Bacteriophages

Bacteriophage applications rely on several core biological features. These include host recognition, life cycle, genome organization, replication behavior, and engineering potential. Their importance varies by application. For therapy, genome safety, lytic activity, persistence, tissue access, and resistance evolution are central. For diagnostics, specificity, reporter compatibility, signal reliability, and matrix performance are more relevant. For biocontrol, the main concerns are stability, host range, delivery mode, and ecological safety. This section therefore summarizes phage biology and engineering from a practical perspective. It focuses on how these features shape downstream therapeutic, diagnostic, and nonclinical biocontrol applications.

### 2.1. Morphology and Genomic Diversity

Bacteriophages are viruses that infect bacteria and usually consist of a protein capsid, infection machinery, and a nucleic-acid genome. In tailed phages, host recognition is mediated not by the tail tube alone but by distal structures such as tail fibers, tail spikes, baseplate-associated proteins, and terminal tail proteins. The older myovirus-, siphovirus-, and podovirus-like terms are now informal morphotypes; most tailed double-stranded DNA phages are classified within Caudoviricetes under genome-based ICTV taxonomy [28,29]. This distinction is important because older morphology-based family names remain common in the literature, whereas current classification is genome centered. It helps separate practical morphological descriptors from formal nomenclature.

Phage genomes span ssRNA, ssDNA, dsRNA, and dsDNA forms, with dsDNA phages dominating described therapeutic and engineering studies. Genome sizes range from small ssRNA phages to large Crassvirales and jumbo phages [30]. Mosaic genome architecture, mobile genetic elements, auxiliary metabolic genes, and receptor-binding modules create a broad design space for host-range adaptation, payload insertion, and functional screening [31,32]. Genomic modularity is also why phages can be repurposed across fields. The same diversity that complicates taxonomy supplies receptor-binding modules, lysis systems, promoters, and packaging signals that can be combined or screened for application-specific functions.

### 2.2. Life Cycles and Infection

Phage infection generally follows lytic or lysogenic/temperate strategies. Lytic phages rapidly replicate and lyse bacterial hosts, making them preferred for direct antibacterial applications [33]. Temperate phages can integrate into host genomes and establish lysogeny through repressor-controlled switches, as illustrated by lambda-like systems and other temperate models [34,35]. For clinical translation, lytic activity must be paired with genome review. A phage that lyses an isolate in vitro may still be unsuitable if it carries genes associated with lysogeny, toxins, or unwanted gene transfer. This screening step is therefore part of candidate selection, not an optional downstream check.

Life-cycle choice is central to biosafety. Environmental factors can influence lysis-lysogeny decisions, and prophages may alter bacterial virulence, serotype, biofilm capacity, or gene-transfer potential [36,37]. Therapeutic development therefore usually prioritizes strictly lytic phages or engineered phages from which lysogeny-associated genes have been removed. For therapeutic development, this distinction matters because lysogenic conversion, transduction, and prophage induction create safety concerns that are irrelevant to many diagnostic or food-surface applications. Lytic biology therefore functions not only as a mechanistic feature but also as a selection criterion for product development.

### 2.3. Phage Host Interactions

Phage–host interaction begins with adsorption to bacterial surface receptors such as lipopolysaccharides, outer-membrane proteins, pili, teichoic acids, or peptidoglycan-associated structures [38,39]. Stable adsorption usually requires conformational changes in distal tail or baseplate structures that trigger genome delivery, so host specificity reflects coordinated receptor-binding machinery rather than a single tail component.

Structural and functional studies of receptor-binding proteins have clarified how phages recognize bacterial hosts. For example, the T5 Pb5-FhuA system reveals receptor-induced conformational change, while polyvalent phages such as GSP004 can recognize different O-antigen receptors across *Salmonella* and *Escherichia coli* [39,40]. Tail-fiber engineering and directed evolution can therefore retarget or broaden host range [41]. Host-range expansion should also be interpreted carefully. A broader host range may improve coverage in a cocktail, but excessive breadth could complicate ecological targeting or increase off-target effects in microbiome applications. The optimal specificity therefore depends on the intended use.

Bacteria counter phage attack through capsules, restriction-modification systems, CRISPR-Cas immunity, abortive-infection modules, and recently described defense systems such as phage anti-restriction-induced system (PARIS) [42,43]. Phages in turn encode counter-defense functions, including anti-CRISPR proteins [44]. This arms race underlies both resistance risk in therapy and the engineering tools used to modify phage genomes.

These host-interaction details are not only mechanistic. They also guide application design. In therapy, receptor use and bacterial defense systems affect host range, resistance evolution, and the durability of phage activity. In diagnostics, the same specificity can support selective recognition and reporter-based detection. In biocontrol, receptor targeting must be weighed against ecological safety and possible non-target effects. Host recognition therefore connects basic phage biology with downstream engineering and validation.

### 2.4. Genome Engineering

Phage genome engineering translates biological knowledge into testable design strategies. Common targets include receptor-binding or tail-fiber genes for host-range tuning, lysogeny-associated genes for safety improvement, and functional insertion sites for reporter genes or antibacterial payloads. The goal is not simply to produce an edited phage, but to improve targeting, biosafety, and functional output for a defined application. Practical engineering decisions should begin with a clear intended use. A reporter phage for diagnostics may prioritize rapid signal output and matrix tolerance, whereas a therapeutic phage must prioritize host-range coverage, genome safety, in vivo persistence, and compatibility with antibiotics. The same genome-editing tool may therefore support very different translational goals.

Major routes include homologous recombination, CRISPR-assisted counterselection, in vitro genome assembly, and genome rebooting [5,45,46,47]. Correctly edited phages must then be evaluated for infectivity, host range, genetic stability, absence of undesirable genes, and application-specific performance. Engineering feasibility alone does not establish therapeutic efficacy. In therapeutic settings, these validation steps should include patient-derived isolates and conditions that approximate the intended infection site. In diagnostics, the same construct should instead be tested for signal-to-noise ratio, speed, live-cell discrimination, and tolerance of blood, food, urine, or environmental matrices.

In CRISPR-assisted workflows, the CRISPR system mainly enriches edited phages by eliminating wild-type genomes. Synthetic biology methods such as Golden Gate assembly, TXTL-based rebooting, and fully cell-free construction accelerate library generation and screening, but still require downstream biological validation [46,47].

#### 2.4.1. Homologous Recombination

Lambda Red recombineering and related homologous recombination methods introduce targeted deletions, insertions, or replacements into phage genomes. They have been applied to lytic phages such as T7 and expanded into multiplex workflows such as SMART, which separates genome modification from later reassembly and rebooting [48,49].

#### 2.4.2. CRISPR Assisted Engineering

**Figure 2:**
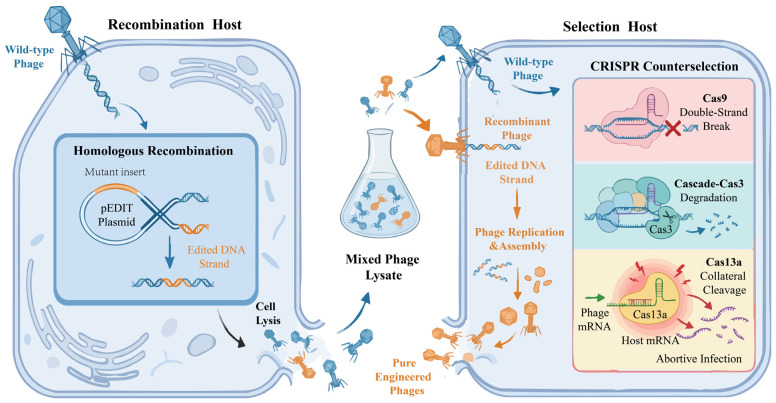
Dual-host model for CRISPR-Cas assisted bacteriophage genome editing and counterselection. A recombination host generates a mixed lysate containing mostly wild-type phages and rare edited recombinants. A selection host expressing a CRISPR-Cas system eliminates wild-type genomes or transcripts while allowing edited phages to replicate. Cas9, Cascade-Cas3, and Cas13a illustrate DNA- or RNA-targeting counterselection routes. The edited region may represent receptor-binding replacement, lysogeny-gene deletion, reporter insertion, or antimicrobial payload insertion, all of which still require experimental validation.

CRISPR-assisted engineering improves recovery of rare recombinant phages by cleaving unedited genomes. Cas9 has enabled efficient editing of staphylococcal phage K [5]. Cas13a-based strategies can be used to edit phages whose DNA is chemically modified or physically protected from DNA-targeting nucleases [50,51], whereas CRISPR-Cas3 has been applied to targeted genome editing of virulent *Pseudomonas* phages [52]. Figure 2 summarizes a dual-host counterselection model in which homologous recombination creates mixed lysates and CRISPR selection enriches edited phages.

RNA-targeting approaches are especially useful when DNA-targeting systems fail. Cas13a has been used to edit T4-like phages with modified bases and the giant phage phiKZ, whose DNA is protected within a nucleus-like compartment [50,51].

#### 2.4.3. Cell Free Assembly and High Throughput Screening

Cell-free and host-independent methods help overcome phage toxicity, host restriction barriers, and slow iterative editing. Yeast artificial chromosome platforms can assemble and modify phage genomes before revival in bacterial hosts, whereas cell-free transcription-translation systems can reboot assembled genomes directly [46,53]. These platforms also make it possible to separate candidate generation from candidate qualification. High-throughput assembly can produce many variants, but only a subset will retain productive infection, acceptable stability, and relevant biological activity. This separation is important because a technically elegant construct may still fail when exposed to patient-derived isolates, complex matrices, or immune clearance.

Golden Gate assembly is particularly useful for ordered reconstruction of phage genomes from multiple fragments. Type IIS restriction enzymes create user-defined sticky ends, allowing one-pot scarless assembly, but internal restriction sites, fragment junctions, cis-acting elements, promoters, and packaging regions must be designed carefully [47,54].

PHEIGES and related TXTL strategies further demonstrate that recombinant phage genomes and host-range libraries can be built and screened entirely in cell-free formats [55]. Barcode-based tracking and bioinformatic tools such as PhagePromoter can support library monitoring and payload-expression design [46,56].

Together, these methods make phages easier to design, build, and screen. Still, technical success is not the final goal. Genome assembly, rebooting, reporter expression, or payload insertion does not prove practical value on its own. Candidate phages still need host-range testing, genome confirmation, safety screening, stability assessment, and functional assays. The tests should fit the intended use. Therapeutic phages need infection-relevant validation. Diagnostic phages need reliable signal output and matrix validation. Biocontrol phages need stability testing, target-specificity assessment, and ecological safety evaluation.

**Figure 3:**
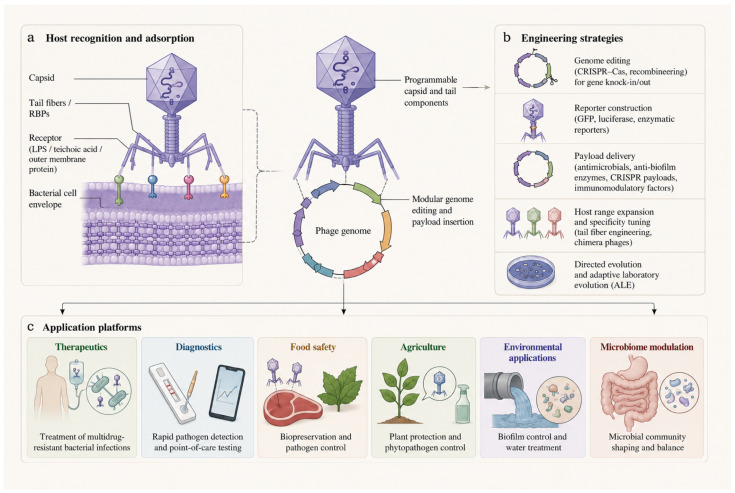
Engineering principles and functional platforms of bacteriophages. The figure summarizes phage host recognition, programmable genome modification, and major application platforms. Receptor-binding proteins determine adsorption to bacterial surface receptors, whereas genome editing, reporter construction, payload delivery, host-range expansion, and directed evolution modify phage function. These engineered and native phages support therapeutics, diagnostics, food biopreservation, plant protection, environmental applications, and microbiome modulation.

Figure 3 summarizes this modular framework. Native and engineered phages link core functional modules with downstream applications. These modules include receptor recognition, genome editing, reporter construction, payload delivery, and directed evolution. They support phage therapy, diagnostics, and nonclinical biocontrol.

## 3. Phage Therapy: Evidence, Persistence, and Resistance-Aware Strategies

### 3.1. Therapeutic Evidence and Clinical Positioning

Clinical exploration of phage therapy began before the antibiotic era, including early typhoid-fever reports from the 1920s and 1940s [57,58]. These observations are historically important because they preceded effective chloramphenicol therapy for typhoid fever [59]. Nevertheless, they lacked randomization, standardized products, blinded outcome assessment, and prespecified microbiological endpoints, so they should not be treated as modern proof of efficacy. The field should therefore avoid a binary interpretation in which phage therapy is either broadly effective or clinically unproven. A more useful question is narrower: for which pathogen, infection site, route, and background therapy does a given phage product provide measurable added benefit? Phage development should therefore be judged against conventional translational pharmacology rather than anecdotal rescue use alone. Historical and modern studies are best presented as a continuum of evidence rather than a single proof base. Early reports explain why the concept persisted, compassionate-use cases show feasibility in urgent settings, and randomized trials are needed to define reproducible efficacy.

In this review, salvage therapy refers to phage use in severe, refractory, or multidrug-resistant infections when standard antimicrobial therapy, source control, or both are ineffective or insufficient. It is a clinical positioning concept rather than a separate regulatory category. This distinction matters because many widely cited phage-therapy reports describe rescue use. Such cases can provide useful clinical and microbiological lessons, but they should not be given the same evidentiary weight as randomized controlled trials.

Modern phage therapy has re-entered clinical research mainly as a response to multidrug-resistant infections. Table 2 summarizes selected randomized trials, compassionate-use reports, personalized inhaled regimens, engineered-phage cases, and adjunctive preclinical studies. Across these examples, the most consistent lesson is that clinical interpretation depends on trial design, product quality, delivery route, pathogen susceptibility, source control, and concurrent antibiotics.

Available evidence is mixed. Compassionate-use and salvage cases can be clinically informative, particularly when conventional treatment options are exhausted, but their evidentiary weight is lower than that of controlled trials. Conversely, randomized studies may show feasibility without clear superiority if dosing, local exposure, target-pathogen burden, or product potency are suboptimal [25,67].

The Patterson case remains a landmark example of personalized phage rescue therapy for disseminated multidrug-resistant *Acinetobacter baumannii* infection [68]. Subsequent genomic analysis showed emergence of phage-tolerant *A. baumannii* during therapy, reinforcing the need for real-time susceptibility testing, genomic monitoring, and adaptive cocktail adjustment [69]. These reports are most useful when interpreted through generalizable translational lessons, including rescue use, resistance emergence, negative trials, and adjunctive randomized evaluation.

Negative or inconclusive clinical studies are equally important. In PhagoBurn, the topical *Pseudomonas aeruginosa* phage cocktail was safe but did not produce the expected efficacy signal, highlighting the importance of titer, formulation stability, and infection-site exposure [25]. Oral coliphages in childhood diarrhea and intravesical phages in urinary tract infection likewise showed that recoverable phage or non-inferiority does not necessarily translate into a clear clinical advantage [67,70]. A further implication is that trial endpoints should be chosen carefully. Microbiological clearance, relapse prevention, inflammatory improvement, organ support, and mortality do not necessarily move together, especially when phages are used with antibiotics and source-control procedures. Future studies should therefore prespecify how microbiological and clinical endpoints will be interpreted together.

Overall, in vitro lytic activity alone is insufficient to predict clinical benefit. Outcomes are shaped by phage-pathogen matching, local pharmacokinetic/pharmacodynamic (PK/PD) relationships, bacterial burden, biofilm penetration, source control, concomitant antibiotic regimens, host immune status, and resistance evolution. Future trials therefore need clinically meaningful endpoints together with microbiological and pharmacological monitoring.

The diSArm study provides preliminary randomized evidence for adjunctive intravenous phage therapy in complicated *Staphylococcus aureus* bacteremia [2]. AP-SA02 was tested with best available antibiotic therapy rather than as monotherapy, and the phase 2a population showed a higher Day 12 clinical response rate with AP-SA02 plus antibiotics than with placebo plus antibiotics [2].

This design is ethically appropriate because withholding effective antibiotics in complicated *S. aureus* bacteremia would be unsafe. At the same time, it limits mechanistic attribution: the study can test added benefit on top of antibiotics, but it cannot establish standalone phage efficacy. Larger trials are needed to define the target population, durability of benefit, safety, and role of phage–antibiotic combinations.

Beyond clinical evidence, therapeutic success also depends on active phage exposure. Phages must persist long enough to reach the infection site, amplify on susceptible bacteria, and avoid rapid host-mediated clearance.

### 3.2. Phage Persistence, Active Exposure, and Immune Clearance

Phage persistence should be distinguished from adaptive anti-phage immunity. During the first several days of treatment, active phage exposure is shaped mainly by innate clearance [71]. Adaptive anti-phage responses may emerge within the first week and may become more relevant during repeated or prolonged dosing, although their timing and magnitude vary according to the phage, route, dose, and host context [66]. Persistence also differs markedly among phages. Experimental findings indicate that blood persistence can vary by three to four orders of magnitude, supporting early screening for high-persistence candidates [72]. High persistence may improve systemic exposure, but it does not remove the need to evaluate route, dose, bacterial burden, tissue distribution, and infection-site PK/PD [71,73].

For clinical translation, host-mediated loss of infectious phages must be considered. Increasing the administered dose alone may not ensure adequate active exposure. Phages may amplify when susceptible bacteria are abundant, decline as bacterial hosts are cleared, or be rapidly inactivated or removed from circulation [71,73]. The administered dose should therefore not be treated as equivalent to active phage exposure. Recoverable infectious phage in blood, infected tissues, or local fluids provides a more relevant pharmacological measure, although circulating titers may not accurately reflect tissue or biofilm exposure [71,73].

After systemic administration, infectious phages may be removed from circulation through several host-mediated mechanisms. Neutrophils, however, appear to contribute only modestly to early clearance in at least one murine model [71]. With repeated or prolonged exposure, phage-specific neutralizing antibodies may develop, accelerate tissue clearance, and reduce therapeutic efficacy [66].

PK/PD-informed dosing should therefore integrate serial measurements of recoverable infectious phages with bacterial-burden dynamics and host immune responses. Modeling studies show that dose, route, bacterial load, and innate immunity jointly shape treatment outcomes [73].

Local delivery may increase infection-site exposure and reduce unnecessary systemic exposure, but it does not eliminate immunological concerns. Neutralizing antibodies have been detected after nebulized phage therapy in patients with cystic fibrosis [74], while a phage–vancomycin hydrogel supported sustained local delivery in a murine fracture-related infection model [75].

### 3.3. Host-Resistance Bypass, Hyper-Aggressive Phages, and Infection Spread

Bacterial resistance to phages can arise through receptor loss or modification, changes in surface polysaccharides, and intracellular anti-phage defense systems. Some phages, however, may delay or bypass the emergence of resistant host populations. This phenotype has been characterized in phage 0524phi7-1, for which no resistant colonies were detected within expanding cleared zones [76]. A related, but not identical, phenotype was generated in the T4-derived variant T4Ar through serial selection on phage-resistant hosts [77].

The hyper-aggressive phage 0524phi7-1 showed several unusual phenotypes, including initially semi-turbid plaques that later cleared, satellite plaques, continued plaque enlargement over several days, and clearing of visible bacterial colonies [76]. These observations accompanied the resistance-bypass behavior described above, but their underlying mechanisms remain unresolved. Proposed explanations include dual-receptor recognition, lysis-inhibition collapse, endolysin release, and reaction–diffusion dynamics [78].

Phage spread may also be important in dense or spatially structured bacterial populations. Progressive plaque enlargement and related spreading phenotypes, as reported for 0524phi7-1 and the T4-derived variant T4Ar, suggest sustained local spread under plaque-assay conditions [76,77,78]. Whether these traits improve therapeutic performance requires validation in infection-relevant models.

Until such validation is available, these phenotypes may inform early phage selection but should not be considered substitutes for personalized cocktails, engineered phages, or phage–antibiotic combinations.

### 3.4. Personalized Cocktails, Engineered Phages, Antibiotics, and Adaptive Monitoring

Personalized cocktails aim to match active phages to patient-derived pathogens and reduce resistance through multireceptor targeting [79,80]. This advantage is greatest when component phages use independent receptors, because simultaneous escape becomes less likely [80]. Dual-receptor Phikzvirus-containing cocktails illustrate how receptor diversity can suppress resistance evolution in *P. aeruginosa* [81].

However, personalization also creates practical burdens. Isolate recovery, phage matching, susceptibility testing, product preparation, quality release, and treatment adjustment must occur within a clinically useful timeframe. Without this infrastructure, personalized therapy may remain limited to specialized centers. Because bacterial populations can shift under phage, antibiotic, and immune pressure, cocktails may require repeated testing and adjustment during treatment.

Cocktail design should not be reduced to simple host-range addition. Component phages may interact synergistically, neutrally, or antagonistically, and optimal ratios require systematic combinatorial testing [82]. For biofilm-associated infections, planktonic assays should be complemented by biofilm and phage–antibiotic validation models [83].

Route selection is also individualized. Intravenous dosing may be needed for systemic infection, inhaled delivery for airway disease, and local injection or implant-associated delivery for orthopedic infection. Early clinical and translational studies support the feasibility of inhaled and local approaches, but each route requires exposure, safety, and matrix-specific validation [84,85].

Engineered phages extend naturally occurring phage activity by adding bactericidal modules, altering host range, removing lysogeny-associated genes, or expressing antibiofilm enzymes [86,87]. These proof-of-concept strategies are scientifically important, but engineered phages have not yet become routine clinical care. They may improve predictability by standardizing payloads, host-range modules, or safety modifications. However, engineering does not remove the need for isolate-level testing. Receptor expression, capsule state, biofilm architecture, and antibiotic exposure can vary across patients and infection sites. Engineering should therefore complement, rather than replace, empirical phagograms.

A central translational gap remains: in vitro engineered activity may not persist in vivo. Receptor accessibility, biofilm barriers, bacterial heterogeneity, and resistance evolution can reduce therapeutic performance [83,88,89], while host clearance and tissue distribution may limit active phage exposure [71,73]. Engineered phages should therefore be treated as optimization platforms, not as intrinsically mature therapeutics. The strongest role for engineered phages may be in situations where a defined functional deficit can be corrected, such as inadequate host range or insufficient biofilm access. Broad claims of superiority over natural phages should be avoided until comparative in vivo data are available.

Examples of engineering value include tail-fiber replacement to alter T7 host specificity [90], deletion of lysogeny-associated repressor genes to generate lytic derivatives [61], and expression of biofilm-degrading enzymes to improve antibiofilm activity [87]. Each strategy still requires validation in the intended pathogen, infection site, and dosing context.

Phage resistance does not always have the same therapeutic consequence. In carbapenem-resistant *K. pneumoniae*, some phage-resistant derivatives showed reduced virulence and increased antibiotic susceptibility [88]. In contrast, phage-selected mutations in *E. coli* O157:H7 increased resistance to both the phage and fosfomycin [91]. Resistance should therefore be evaluated as an evolutionary trade-off rather than as a uniformly harmful or beneficial outcome.

Phage–antibiotic synergy (PAS) is context-dependent rather than automatic. Its outcome depends on the antibiotic mechanism of action, relative concentrations, bacterial physiology, and phage characteristics [92]. Therefore, combinations should be selected through mechanism-guided screening, time-kill assays, susceptibility testing, and relevant biofilm models rather than empirical pairing alone. Because PAS can be antagonistic as well as synergistic, antibiotic choice should be treated as part of phage selection [92]. The most rational combinations are those that either enhance bacterial killing [93,94] or direct resistance evolution toward reduced fitness, lower virulence, or restored antibiotic susceptibility [88,94].

Biofilm-associated infections require infection-site-specific validation. In a sheep sinusitis model, topical phage treatment with or without EDTA reduced *Staphylococcus aureus* biofilm burden [95]. However, these findings should not be directly extrapolated to deep-seated or implant-associated infections.

Resistance management should continue during therapy. When bacterial burden rebounds, isolates should be retested for phage and antibiotic susceptibility. Genome sequencing may help identify receptor mutations, defense-system changes, or antibiotic-resistance alterations when feasible. Multicenter and case-level evidence indicates that serial microbiological reassessment can help interpret response, relapse, or failure and guide treatment adjustment [96,97].

Taken together, effective phage therapy requires active-exposure monitoring, route and formulation optimization, receptor-guided phage selection, mechanism-validated phage–antibiotic combinations, biofilm-aware testing, and adaptive resistance surveillance. These measures may improve durability in complex infections and support more precise clinical use. This adaptive model makes phage therapy closer to precision antimicrobial management than to a fixed product prescription. It also creates a need for microbiology laboratories capable of rapid phage susceptibility testing, repeated isolate recovery, and communication with treating teams during the treatment course.

## 4. Diagnostics and Biosensing

### 4.1. Phage Typing and Bacterial Identification

Phages have long been used for bacterial identification because their lysis patterns can distinguish strains. Traditional phage typing contributed to outbreak investigation, as shown in hospital *Staphylococcus aureus* surveillance [98]. Modern platforms increasingly replace plaque-pattern interpretation with defined receptor-binding proteins, reporter phages, and quantitative readouts.

**Figure 4:**
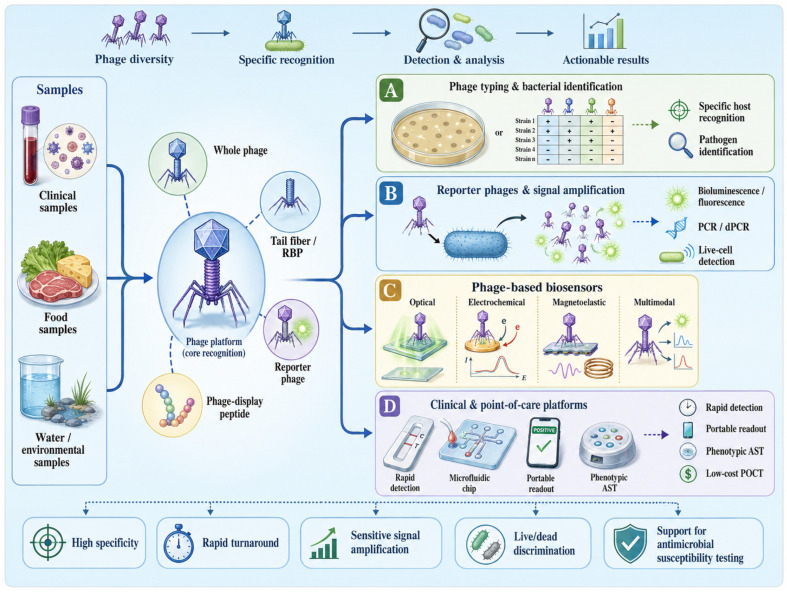
Overview of phage-based diagnostic and biosensing platforms. Whole phages, receptor-binding proteins, reporter phages, and phage-display-derived peptides provide recognition or signal modules for clinical, food, and environmental samples. These modules support phage typing, reporter-phage amplification, optical/electrochemical/magnetoelastic biosensors, lateral-flow assays, microfluidics, portable readers, and phenotypic antimicrobial susceptibility testing. Major advantages include specificity, rapid readout, sensitive amplification, live/dead discrimination, and compatibility with point-of-care formats.

Receptor-binding proteins provide highly specific capture elements. A paper-based analytical device using a phage tail-fiber protein detected *Pseudomonas aeruginosa* and supported antimicrobial susceptibility testing in serum, urine, and milk matrices [4]. Figure 4 summarizes the main diagnostic routes, from typing to reporter phages, biosensors, and point-of-care workflows.

### 4.2. Reporter Phages and Signal Amplification

Reporter phages carry detectable genes such as nluc or gfp and report viable target bacteria after infection. CRISPR-Cas9 engineering of staphylococcal phage K carrying nluc enabled live *Staphylococcus aureus* detection in whole blood and raw milk within hours while preserving live/dead discrimination [5].

Phage-mediated amplification can also use phage DNA as a PCR-amplifiable tag. In aflatoxin B1 detection, nanobody phage-mediated qPCR and digital PCR achieved ultrasensitive quantification in agricultural products, illustrating how phage-derived signal amplification can extend beyond bacterial targets [99].

### 4.3. Phage-Based Biosensors

Phage-based biosensors immobilize intact phages or receptor-binding proteins on transducer surfaces. Binding of target bacteria is then converted into optical, electrical, mass-based, or multimodal signals. Practical performance depends on recognition specificity, immobilization stability, matrix interference, readout device, and validation setting [100,101]. For diagnostics, the main advantage of phage specificity is not merely recognition of a bacterial species. In some formats, phage infection provides evidence of viability, which can help distinguish live pathogens from residual nucleic acids or dead cells. This feature is particularly valuable when rapid decisions about antimicrobial susceptibility, contamination control, or decolonization are needed.

#### 4.3.1. Optical Biosensing

Optical biosensors use fluorescence, bioluminescence, surface plasmon resonance, or related signal modes. Representative systems include a dual fluorescence/bioluminescence assay that distinguishes live and total *Salmonella typhimurium* and an SPR platform using a lambda tail-protein fragment for label-free *E. coli* detection [100,101]. In practice, biosensor performance should be reported with matrix, enrichment time, sample volume, and total workflow time, not only with the lowest analytical limit of detection. This improves comparability across platforms and prevents overinterpretation of idealized analytical conditions.

#### 4.3.2. Electrochemical Biosensing

Electrochemical sensors quantify bacterial capture through changes in current, impedance, or electrochemiluminescence signals. Phage-immobilized platforms have enabled sensitive detection of *Salmonella typhimurium* and *E. coli* O157:H7 in food matrices [102,103]. A dual-mode ECL/EIS sensor has also been developed for *Pseudomonas aeruginosa* detection in PBS and artificial urine [104].

#### 4.3.3. Magnetoelastic Biosensing

Magnetoelastic biosensors detect bacterial capture as a mass-induced resonance-frequency shift. This non-optical readout is useful for opaque or matrix-rich samples. A planar spiral coil-based magnetoelastic sensor with humidity-resistant phages enabled simultaneous detection of *Salmonella typhimurium* and *E. coli* O157:H7 on fresh produce [105].

#### 4.3.4. Multimodal Biosensing

Multimodal strategies combine phages with aptamers, DNAzymes, microfluidics, or encoded probes to improve reliability and reduce false-positive readouts. Recent dual-mode platforms detected multiple foodborne pathogens with electrochemical and fluorescence outputs and short sample-to-result times [106,107].

Overall, phage biosensors can achieve high sensitivity and specificity, but practical translation requires real-sample validation, robust immobilization chemistry, matrix-tolerant workflows, and clear comparison with established diagnostic standards. Another issue is workflow realism. A platform with an excellent limit of detection may still be difficult to use if it requires complex sample preparation, unstable reagents, expensive readers, or expert interpretation. For clinical adoption, phage-based diagnostics should be evaluated against the speed, cost, and robustness of culture, PCR, MALDI-TOF, and established lateral-flow platforms.

### 4.4. Clinical and Point-of-Care Platforms

Point-of-care translation requires rapid sample preparation, simple readout, low cost, and compatibility with decentralized testing. Phage-derived technologies contribute recognition elements, signal templates, and peptide binders that can be integrated with lateral-flow, microfluidic, smartphone-readable, or portable optical platforms. POCT applications are also constrained by operational context. A field food-safety test may prioritize portability and low cost, whereas an emergency clinical test may prioritize sensitivity, turnaround time, and integration with antimicrobial decision-making.

For infectious disease diagnostics, phage-templated hierarchical plasmonic assembly has enabled surface-enhanced Raman lateral-flow immunoassays with strong signal amplification, and M13-based chemiluminescent lateral-flow formats have supported smartphone-readable detection of SARS-CoV-2 nucleoprotein [108,109].

Phage-associated enzymes can also support nucleic-acid diagnostics. Recombinase polymerase amplification based on the T4 UvsX system operates at mild temperatures, and recent work on phase-separation-controlled recombination has improved CRISPR-compatible RNA detection efficiency [110].

For biomarker testing, phage-display-derived affinity peptides offer stable, low-cost alternatives to antibodies. A CEA-binding peptide conjugated to gold nanoparticles was integrated into an immunochromatographic strip and validated in clinical serum samples [111].

The platforms summarized in Table 3 differ in target, recognition element, readout, limit of detection, sample matrix, and time to result. For this reason, analytical sensitivity alone should not be used as the only measure of practical diagnostic value.

## 5. Nonclinical Applications

### 5.1. Food Biopreservation

Foodborne pathogens remain a major public-health and economic problem, and phages provide a natural biopreservation strategy because they can target bacterial contaminants without broad chemical preservation. Commercial and regulatory experience is most mature for products targeting *Listeria*, *Salmonella*, Shiga toxin-producing *E. coli*, and *Shigella* (Table 1). The strongest food applications are those in which phages are applied to surfaces or matrices where target bacteria are accessible and where product claims can be validated through controlled challenge testing. Complex foods with uneven surfaces, fat-rich matrices, or protective microenvironments may require higher doses, repeated application, or combination with other hurdles.

Commercial preparations such as ListShield and SalmoFresh illustrate the regulatory progress and practical use of phage-based food biocontrol [112,113,114,115,116]. Their efficacy, however, remains matrix dependent and is influenced by temperature, packaging conditions, application mode, and host range. Integration into existing food-processing workflows without compromising product quality is therefore important for wider adoption.

Hurdle strategies can further improve performance. Combining phage P100 with bacteriocin AS-48 reduced *L. monocytogenes* in fish products more effectively than either treatment alone [117]. Industrial deployment, however, requires matrix-specific dosing, manufacturing control, formulation stability, regulatory alignment, consumer acceptance, and ecological safety assessment [118,119].

### 5.2. Agriculture and Plant Protection

In agriculture, phages provide targeted biocontrol against bacterial plant pathogens. Field and formulation studies support activity against bacterial spot of tomato and pepper, and AgriPhage was registered by the US EPA as a phage biopesticide for plant disease control [7,120]. Phage cocktails have also been developed against fire blight caused by *Erwinia amylovora* [121]. Agricultural deployment is more ecologically open than food-surface treatment. Phages released onto plants or soils encounter ultraviolet light, desiccation, fluctuating temperature, microbial competition, and spatial heterogeneity. Formulation, timing, and compatibility with existing disease-management programs therefore strongly affect field performance.

Beyond direct pathogen lysis, phages may also influence plant-associated microbiomes. Rhizosphere phage communities can contribute to the suppression of bacterial wilt, while phage treatment of pear flowers can reduce *Erwinia amylovora* with limited disruption of the resident microbiome [122,123]. Plant protection also raises formulation challenges. Ultraviolet protection, adhesion to plant surfaces, rainfastness, and application timing may determine whether laboratory activity translates into reliable field control.

### 5.3. Fermentation and Environmental Engineering

In fermentation, phage contamination can lyse starter or production bacteria and cause process failure, particularly in dairy and microbial manufacturing systems [124,125]. Control strategies are shifting from physical exclusion alone toward resistant starter development, adaptive evolution, and transferable CRISPR-Cas immunity, as shown for *Lactococcus lactis* [126].

In environmental engineering, phages can be used to manage biofilms, membrane fouling, and sludge bulking. Examples include phage-mediated restoration of membrane bioreactor flux and lysis of *Haliscomenobacter hydrossis* to improve sludge settling [127,128]. Fermentation settings differ from therapy because the goal is often preventing phage activity rather than using it. This contrast highlights that phages can be either a product platform or a process hazard, depending on context.

### 5.4. Microbial Ecology and Ecological Effects

Phages also shape microbial ecology by lysing dominant bacterial hosts, maintaining lineage diversity, and mediating horizontal gene transfer [37,129,130]. These effects can influence gut, soil, sewer, and plant-associated communities, but the direction of change depends on local host availability and network structure [131,132]. Ecological interpretation should remain balanced. Phages can suppress pathogens and stabilize diversity, but they can also reshape communities in unexpected ways. The relevant safety question is not whether phages have ecological effects, but whether those effects are predictable, monitored, reversible, and acceptable for the intended deployment scale.

Ecological risk therefore requires explicit assessment. Phages may carry or mobilize antibiotic-resistance genes, although wastewater studies suggest their contribution to the resistome can be limited in some systems [133]. Long-term deployment should include metagenomic monitoring, non-target community assessment, and environmental traceability.

## 6. Translation and Future Directions

**Figure 5:**
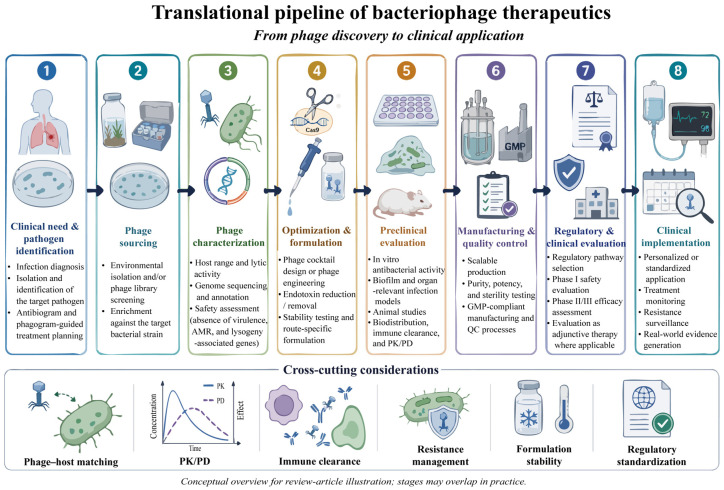
Translational pipeline of therapeutic bacteriophages. The figure outlines steps from clinical need and pathogen identification to phage development, manufacturing, regulatory evaluation, and clinical implementation. Cross-cutting considerations include phage–host matching, PK/PD, immune clearance, resistance management, formulation stability, biosafety, and regulatory standardization. These factors require continuous evaluation during development and clinical use.

Phage technologies are moving from discovery-driven research toward translational platforms, but implementation requires an integrated framework linking patient-derived pathogen identification, phage screening, formulation, manufacturing, quality control, regulatory review, clinical monitoring, and post-treatment resistance surveillance. Figure 5 summarizes this translational pipeline and emphasizes that manufacturing, biosafety, regulation, ethics, and intelligent design are not late-stage add-ons. They are cross-cutting requirements that influence phage selection, product preparation, trial design, and clinical or environmental deployment.

### 6.1. Manufacturing and Quality Control

Industrial-scale phage production must deliver consistent potency, purity, stability, and safety under quality-controlled conditions [134]. Upstream fermentation requires optimization of host physiology, inoculum density, multiplicity of infection, culture conditions, and harvest timing to maximize titer and reproducibility [135,136]. Manufacturing standards also differ by application. Intravenous therapeutic products require stricter endotoxin, sterility, identity, and potency controls than food-processing aids or environmental biocontrol preparations. Nevertheless, all applications require a defined relationship between production process, product composition, and claimed performance.

For Gram-negative hosts, downstream purification must remove endotoxin because crude lysates can contain inflammatory lipopolysaccharide. Membrane-based methods, including ultrafiltration and tangential flow filtration, can be used alongside anion-exchange chromatography for downstream concentration and purification [134,137]. Residual endotoxin should be quantified separately using a validated assay, such as the limulus amebocyte lysate test [134]. Endotoxin control is particularly important because it can confound interpretation of both safety and efficacy. Inflammatory reactions caused by contaminants may be mistaken for phage-related immunogenicity or infection progression if purification and release testing are inadequate.

Formulation stability is another barrier. Phages can be inactivated by temperature, pH, drying, and shear stress, so lyophilized powders, cryoprotectants, spray-dried microparticles, and composite formulations are being developed to extend shelf life and support pulmonary, oral, or local delivery [138,139].

Personalized therapy adds a speed constraint: patient isolates must be matched rapidly to active phages, prepared under an acceptable quality framework, and retested if resistance emerges. Experience from Eliava and UCSD/IPATH illustrates the value of curated phage libraries, standardized phagograms, and flexible adjustment workflows [140,141].

### 6.2. Regulation and Ethics

Regulation must balance individualized access with product quality, safety, and accountability. Depending on the jurisdiction, phages may be regulated as magistral preparations, investigational products, marketed preparations, or conventional medicinal products, or accessed through compassionate-use or therapeutic-experimentation pathways. Regulatory frameworks should define responsibility for phage selection, genome review, manufacturing release, clinical administration, outcome monitoring, and adverse event reporting. This is especially important when cocktails are adjusted during treatment or patient-specific preparations are used. Distinct standards may therefore be needed for standardized off-the-shelf cocktails and patient-specific preparations.

Table 4 summarizes representative regulatory and clinical-access models for selected countries and regions, including pharmacy-based magistral preparation, expanded access, special-access schemes, compassionate authorization, marketed country-specific products, academic therapeutic-experiment frameworks, and general drug-development pathways.

These examples show that there is no single global regulatory model for phage therapy. The common challenge is to reconcile rapid patient-specific access with reproducible manufacturing, defined release testing, safety-supporting evidence, and clear responsibility for clinical monitoring and adverse-event reporting.

GMP constraints are also an access issue. Dedicated facilities and outsourced production can be expensive, limiting compassionate-use capacity and pushing many cases toward academic or non-GMP production [156]. This creates a tension between clinical urgency and fully compliant manufacturing.

Trial ethics are similarly application-specific. In severe drug-resistant infections, withholding effective antibacterial therapy is unacceptable, so investigational phages are often evaluated as adjuncts to standard or best available antibiotic therapy rather than as replacements. Trial design should also account for rapid bacterial evolution. Protocols that permit isolate re-sampling and predefined adaptation rules may better reflect real phage use than rigid regimens, but they also require careful statistical and regulatory planning.

The AP-SA02 diSArm design illustrates this point: both arms received best available antibiotic therapy, while the investigational arm received adjunctive phage treatment [2]. Such designs protect participants but make it harder to separate phage effects from antibiotic effects or phage–antibiotic interactions.

When conventional randomized designs are difficult, standardized monitoring frameworks such as STAMP may support systematic safety and tolerability assessment while preserving participant protection [147]. Future trials should explicitly state whether phages are being tested as adjunctive, replacement, or salvage therapy.

Equitable access remains unresolved. High customization costs, limited production capacity, and long treatment delays can make phage therapy dependent on location, institutional resources, or ability to pay, raising concerns about distributive justice [3,156]. Access considerations should be addressed early rather than after proof of concept. A technology that depends on rare phage banks, expensive custom manufacturing, and prolonged screening may help individual patients but remain difficult to scale. Sustainable models will need shared libraries, transparent quality standards, and reimbursement pathways.

### 6.3. Safety and Biosafety

Safety assessment must extend beyond acute toxicity to immune responses, genomic risk, microbiota disturbance, and ecological effects. These risks differ by route, dose, phage type, host bacterium, and treatment duration. Safety monitoring should be proportional to risk. Short topical use in food or local wound settings does not raise the same questions as repeated intravenous dosing in immunocompromised patients. Conversely, environmental release may require ecological surveillance even when direct human toxicity is unlikely.

Immune monitoring is important because phage exposure can induce phage-specific antibodies, neutralization, memory responses, and accelerated clearance, particularly with repeated dosing [66]. These responses may reduce efficacy even when the target bacterium remains phage-susceptible.

Genomic safety assessment requires whole-genome sequencing to screen for lysogeny-associated markers, toxin and virulence genes, and antimicrobial-resistance genes [157,158]. Automated tools such as Sphae can support candidate screening, but putative markers, including integrases, recombinases, and transposases, should be interpreted in their genomic context through expert review and quality-controlled annotation [158].

Microbiota effects should be evaluated with longitudinal 16S rRNA sequencing or metagenomics when relevant. Compared with broad-spectrum antibiotics, targeted phages may spare non-target commensals, but sustained ecological safety must be demonstrated for each use case [159,160].

### 6.4. AI-Driven Precision Phages and Cross-Platform Integration

AI-driven precision-phage strategies may accelerate phage–host matching, cocktail prioritization, and regional surveillance-informed design [161,162]. Current prediction models, however, remain imperfect and are influenced by dataset source, taxonomic level, and evaluation strategy. AI may be most useful when coupled to continuously updated surveillance datasets. Regional bacterial genomics, resistance profiles, and phage–host interaction data could help prioritize phage-bank expansion and cocktail composition. However, biased datasets and sparse negative interaction data remain major limitations.

Computational tools should therefore narrow the experimental search space rather than replace phagograms, plaque assays, host-range determination, genome-based safety screening, formulation testing, and application-specific functional validation [161,162]. Accordingly, AI, CRISPR, and synthetic biology should be considered accelerators rather than substitutes for biological testing or clinical evidence.

Beyond AI-driven candidate selection, integrating phages with complementary modalities may further expand the therapeutic design space. Phage–antibiotic cocktails may broaden antibacterial coverage and improve activity against biofilm-associated bacteria, whereas phage–silver nanoparticle constructs may enhance antibiofilm effects [163,164]. These strategies remain context dependent and require validation against the intended pathogen and in the relevant application setting.

## 7. Conclusions

Bacteriophages have re-emerged as versatile biological platforms for therapy, diagnostics, food safety, agriculture, fermentation control, environmental engineering, and microbiome modulation. Their renewed relevance reflects both the AMR crisis and the expanding ability to engineer phage genomes, receptor-binding structures, reporter systems, and functional payloads. The current evidence, however, argues against treating phage technology as a single uniform field. Therapeutic phages, diagnostic platforms, and nonclinical biocontrol products differ in validation requirements, safety concerns, regulatory pathways, and practical endpoints.

For therapy, available data are promising but uneven. Compassionate-use cases, personalized treatments, preclinical models, and early randomized trials support feasibility, especially as adjunctive treatment for difficult multidrug-resistant infections. Yet in vitro lysis, successful engineering, or isolated clinical improvement cannot establish routine efficacy. Translation will require standardized phagograms, active-phage exposure monitoring, PK/PD-informed dosing, bacterial-burden dynamics, resistance tracking, biofilm-aware testing, and clinically meaningful endpoints.

Diagnostics and biosensors appear closer to platform standardization, but their value depends on real-sample validation, matrix tolerance, reproducible sample-to-result workflows, instrument requirements, cost, and comparison with existing standards. Food, agricultural, fermentation, and environmental uses are more practically established in some settings, but long-term ecological safety, non-target effects, horizontal gene transfer, environmental persistence, and resistance emergence must remain central to governance.

Overall, bacteriophages offer highly specific, programmable, and ecologically informed solutions to urgent biomedical and industrial problems. Their future impact will depend less on theoretical versatility than on reproducible, safe, regulatable, ethically acceptable, and economically accessible implementation pathways. A mature phage field will require not only better phages, but better standards for evidence, manufacturing, regulation, and long-term safety assessment.

## Figures and Tables

**Table 1:** Representative approved, marketed, and clinical-stage bacteriophage products across therapeutic and nonclinical applications.

Category	Product/Candidate	Target Pathogen	Regulatory/Clinical Stage/Status	Application	Refs.
Food Safety	ListShield™	*Listeria monocytogenes*	FDA GRAS; GRN 528; food additive regulation 21 CFR §172.785	Food-surface application	[8]
	SalmoFresh™	*Salmonella* spp.	FDA GRAS; GRN 435; USDA FSIS Directive 7120.1	Poultry, seafood, meat, produce	[9]
	EcoShield™/EcoShield™ PX	Shiga toxin-producing *Escherichia coli* (STEC), including *E. coli* O157:H7	FDA GRAS; FCN 1018; GRN 834; expanded use for grain and grain products	Food-surface/processing application	[10]
	ShigaShield™	*Shigella* spp.	FDA GRAS; GRN 672	Food-surface application	[11]
	PhageGuard Listex™/PhageGuard L™	*Listeria monocytogenes*	FDA GRAS; GRN 218; FSANZ/Health Canada/other processing-aid approvals	Food-processing aid	[12]
	PhageGuard S™	*Salmonella* spp.	FDA GRAS; GRN 468; FSANZ/Health Canada/other processing-aid approvals	Food-processing aid	[12]
	PhageGuard E™	*Escherichia coli* O157:H7/STEC	FDA GRAS; GRN 757/USDA-recognized processing-aid status reported by developer	Food-processing aid	[12]
	OvaPhage	Enterobacteriaceae including *Salmonella* spp.	Health Canada Letter of No Objection (LONO) reported by developer, 2025	Egg-surface application	[13]
	PhageFend	*Salmonella enterica*	Health Canada Letter of No Objection (LONO) reported by developer, 2025	Raw chicken breast/food-surface application	[13]
	SalmoLyze™	*Salmonella* spp.	FDA Center for Veterinary Medicine (CVM) approval for pet-food use, 2025	Pet-food processing aid	[14]
Agricultural/Veterinary	AgriPhage™	*Xanthomonas campestris* pv. vesicatoria; *Pseudomonas syringae* pv. tomato	US EPA registered pesticide; EPA Reg. No. 67986-1	Foliar/chemigation crop application	[15]
	BAFASAL^®^	*Salmonella* Enteritidis; *Salmonella* spp.	EU feed-additive authorization; Commission Implementing Regulation (EU) 2025/1390	Authorized for use in water for drinking only; not for use in complementary feed (applicant withdrew request April 2025)	[16]
Human Therapeutic Phage Products	AP-SA02	*Staphylococcus aureus* (MSSA/MRSA)	Phase 1b/2a randomized data reported; Phase 3 planned; FDA Fast Track designation reported by developer	Intravenous adjunctive therapy with best available antibiotics	[2,17]
	AP-PA02	*Pseudomonas aeruginosa*	Phase 1b/2a completed; Phase 2 Tailwind study completed with topline results reported by developer	Inhaled phage product for chronic lung infection	[18]
	SNIPR001	*Escherichia coli*	Phase 1 completed/published; follow-on clinical development ongoing	Oral CRISPR-Cas-armed phage cocktail	[19]
	LBP-EC01	*Escherichia coli*	Phase 2 ELIMINATE trial; dose-finding data published	Intravenous recombinant/engineered phage cocktail	[20]
	BX004/BX211	*Pseudomonas aeruginosa*; *Staphylococcus aureus*	BX004: Phase 2b trial discontinued in December 2025 after internal review and DMC feedback reported by developer; BX211: Phase 2 diabetic foot osteomyelitis data reported by developer	Nebulized/local phage products	[21,22]
	Sextaphage^®^ polyvalent pyobacteriophage	*Staphylococcus*, *Streptococcus*, *Proteus*, *Klebsiella*, *Pseudomonas aeruginosa*, *Escherichia coli*	Commercially marketed/registered phage preparation in Russia	Oral, local, and external solution	[23]
	Phago-Pyo bacteriophage	*Staphylococcus*, *Streptococcus*, *Escherichia coli*, *Pseudomonas aeruginosa*, *Proteus* spp.	Commercially available paratherapeutic phage product in Georgia under a country-specific framework	Oral, local, external, catheter-based, or rectal route depending on indication	[24]
	Phago-Intesti bacteriophage	*Shigella*, *Salmonella*, *Escherichia coli*, *Proteus*, *Staphylococcus*, *Pseudomonas aeruginosa*, *Enterococcus*	Commercially available paratherapeutic phage product in Georgia under a country-specific framework	Oral and rectal use according to product information	[24]
	PP1131 (PhagoBurn)	*Pseudomonas aeruginosa*	Randomized controlled phase 1/2 trial completed; trial terminated early	Topical phage cocktail for burn-wound infection	[25]
	AB-SA01	*Staphylococcus aureus*	Phase 1 trial completed/published	Intranasal irrigation	[26]
	TP-102	*Staphylococcus aureus*, *Pseudomonas aeruginosa*, *Acinetobacter baumannii*	Phase I/IIa completed/published; Phase IIb development reported	Topical phage cocktail for diabetic foot ulcers	[27]

Note: This table provides a representative overview rather than an exhaustive list. The listed products and candidates differ in intended use, regulatory pathway, formulation, route of administration, and level of supporting evidence. For this reason, regulatory recognition in food safety, agricultural or veterinary applications, or country-specific therapeutic frameworks should not be interpreted as equivalent to randomized clinical evidence for human therapeutic efficacy. GRAS, generally recognized as safe; LONO, Letter of No Objection; EPA, Environmental Protection Agency.

**Table 2:** Selected clinical and translational studies of bacteriophage therapy.

Indication/Infection Context	Target Pathogen	Study Type/Route	Key Takeaways	Refs.
Burn wound infection	*Pseudomonas aeruginosa*	Randomized, controlled, double-blind phase 1/2; topical	PP1131 was generally tolerated but reduced bacterial burden more slowly than standard care. Loss of phage titer resulted in markedly lower-than-planned dosing.	[25]
Cystic fibrosis (MDR/PDR airway infection)	MDR/PDR *P. aeruginosa*	Compassionate-use case series (*n* = 9); personalized nebulized therapy	Treatment was associated with lower sputum bacterial burden and improved lung-function measures, with no reported adverse events. The uncontrolled design limits efficacy inference.	[60]
Drug-resistant mycobacterial disease (multi-case series)	Drug-resistant mycobacteria	Compassionate-use series (20 patients); intravenous and/or aerosolized	No adverse reactions were attributed to phage therapy, and 11 patients showed favorable clinical or microbiological responses. Neutralizing antibodies developed in some intravenously treated patients.	[3]
Disseminated M. abscessus after lung transplant (landmark case)	*Mycobacterium abscessus*	Case report; intravenous three-phage cocktail containing engineered lytic derivatives	Intravenous treatment was well tolerated and was associated with objective clinical improvement. The single-patient design limits definitive efficacy attribution.	[61]
VRE infection in pediatric transplant recipient	VRE *Enterococcus faecium*	Case report; intravenous magistral two-phage preparation	Treatment was associated with reduced inflammatory markers and clinical improvement, with no reported treatment-related clinical adverse events. Causal attribution remains limited.	[62]
Prosthetic joint infection (MRSA), salvage setting	MRSA	Salvage case report; adjunctive intraoperative and intravenous phage therapy	Sterile subsequent cultures and no clinical recurrence at 11 months were reported, but concurrent surgery and antibiotics limit attribution to phage therapy.	[63]
Pandrug-resistant A. baumannii bacteremia	Pandrug-resistant *A. baumannii*	Preclinical in vitro and murine bacteremia study; intraperitoneal phage ± ciprofloxacin	ΦAb4B reduced bacterial burden. Phage plus ciprofloxacin yielded 92% survival versus 67% with phage alone, but the difference was not statistically significant. Higher or booster dosing provided no consistent benefit.	[64]
Ventilator-associated pneumonia (VAP) adjunctive strategy	*P. aeruginosa*	Murine VAP model; phage cocktail + meropenem	Adjunctive phage therapy accelerated clinical improvement and reduced lung epithelial injury in the murine VAP model. Complementary cell experiments showed a lower effective meropenem concentration and limited resistance emergence.	[65]
Immune responses limiting repeat dosing (translation risk)	Vancomycin-resistant *E. faecalis*	Murine intestinal colonization and re-exposure model; repeated intraperitoneal dosing	Repeated exposure induced neutralizing antibodies, accelerated tissue clearance, and reduced the efficacy of the same phage cocktail upon reuse.	[66]

**Table 3:** Performance comparison of representative phage-based and phage-derived diagnostic and biosensing platforms discussed in Section 4.

Platform/Readout	Target	Key Performance and Validation Setting	Refs.
Section 4.1; Paper-based analytical device with fluorescence readout	*Pseudomonas aeruginosa*	Recognition/signal element: Phage tail fiber protein as capture element; AMP-FITC as signal probe; Analytical performance: Linear range: 1.0 × 10^3^–1.0 × 10^7^ CFU/mL; LOD: 137 CFU/mL; Validation/time: Serum, urine, and milk samples; AST results compared with broth dilution; On prefabricated PAD: 40 min bacterial incubation plus 6 min signal-probe incubation; AST workflow included 2 h antibiotic exposure before PAD detection	[4]
Section 4.2; Bioluminescent reporter phage assay	Viable *Staphylococcus aureus*	Recognition/signal element: Engineered staphylococcal phage K carrying nluc; Analytical performance: Growth medium: 707–2617 CFU/mL; whole human blood: 136–2151 CFU/mL; bovine raw milk: 55–514 CFU/mL, depending on strain and matrix; Validation/time: Growth medium, whole human blood, and bovine raw milk; Reporter signal measured after 3 h phage infection; complex-matrix workflow included 1 h pre-infection incubation/stimulation	[5]
Section 4.2; Phage-mediated qPCR/dPCR amplification assay	Aflatoxin B1	Recognition/signal element: Nanobody-displaying phage as PCR-amplifiable signal tag; Analytical performance: Phage-qPCR LOD: 0.093 ng/mL; phage-dPCR LOD: 0.015 ng/mL; phage-dPCR linear range: 0.063–8.837 ng/mL; Validation/time: Corn, flour, soy sauce, and other agricultural products; Total assay time less than 2 h	[99]
Section 4.3.1; Dual-mode fluorescence/bioluminescence biosensor	Live and total *Salmonella typhimurium*	Recognition/signal element: Magnetic phage capture probe and fluorescent phage signal tag; Analytical performance: 55 CFU/mL for total cells; 9 CFU/mL for live cells; Validation/time: Milk samples; also tested in water samples; Within 20 min	[100]
Section 4.3.1; SPR biosensor	*Escherichia coli* K-12	Recognition/signal element: Fragment of lambda phage tail protein J; Analytical performance: Detection range: 2 × 10^4^–2 × 10^9^ CFU/mL; LOD: 2 × 10^4^ CFU/mL; Validation/time: Laboratory bacterial suspension; Within 20 min	[101]
Section 4.3.2; Electrochemical DPV biosensor	*Salmonella typhimurium*	Recognition/signal element: Immobilized *S. typhimurium*-specific phage particles on BC/Ppy/RGO substrate; Analytical performance: 1 CFU/mL in PBS; 5 CFU/mL in milk; 3 CFU/mL in chicken; Validation/time: PBS, milk, and chicken; Optimized response time of 30 min	[102]
Section 4.3.2; Label-free electrochemical biosensor	*E. coli* O157:H7 GXEC-N07	Recognition/signal element: Phage EP01 immobilized on CFGO/CB-modified glassy carbon electrode; Analytical performance: Linear range: 10^2^–10^7^ CFU/mL; LOD: 11.8 CFU/mL; Validation/time: Fresh milk and raw pork; <30 min	[103]
Section 4.3.2; Dual-mode ECL/EIS electrochemical sensor	*Pseudomonas aeruginosa*	Recognition/signal element: *P. aeruginosa* phage M-PaP1 immobilized on MWCNT-COOH-modified screen-printed electrode; Analytical performance: ECL LOD: 0.755 CFU/mL; linear range: 2.28–10^10^ CFU/mL; Validation/time: PBS and artificial urine; Complete sample-to-result time not clearly reported in the full text	[104]
Section 4.3.3; Signal-enhanced magnetoelastic biosensor	*Salmonella typhimurium* and *E. coli* O157:H7	Recognition/signal element: Humidity-resistant wild-type tailed phages immobilized on ME sensors; Analytical performance: For simultaneous detection on apple: 1.7 ± 0.4 log CFU/25 mm^2^ for *S. typhimurium*; 1.6 ± 0.3 log CFU/25 mm^2^ for *E. coli* O157:H7; Validation/time: Apple surface/fresh produce surface; direct surface testing without sample preparation; Optimized phage-binding exposure time: 16 min; performance testing used 20 min incubation on contaminated apple surfaces	[105]
Section 4.3.4; Microfluidic dual-mode biosensor	*E. coli*, *S. typhimurium*, and *S. aureus*	Recognition/signal element: Phage-modified magnetic bead capture probes and aptamer/DNAzyme/ferrocene-functionalized COF signal tags; Analytical performance: Linear range: 10–10^7^ CFU/mL; LODs: 4 CFU/mL for *E. coli*, 8 CFU/mL for *S. typhimurium*, and 9 CFU/mL for *S. aureus*; Validation/time: Milk, vegetables, pork, and fish samples; Entire sample processing and detection process within 40 min; workflow included 20 min bacterial capture and 10 min fluorescence reaction	[106]
Section 4.3.4; Tesla valve-assisted dual-mode biosensor	*E. coli* O157:H7 and *S. typhimurium*	Recognition/signal element: Phage/DNAzyme co-modified ZIF-8 encoded probes; Analytical performance: Linear range: 10–10^7^ CFU/mL; LODs: 5 CFU/mL and 8 CFU/mL, respectively; Validation/time: Milk, pork, fish, and lettuce samples; Within 30 min	[107]
Section 4.4; SERS-LFIA	SARS-CoV-2 IgM and IgG antibodies	Recognition/signal element: MS2 phage-templated hierarchical plasmonic assembly; Analytical performance: IgM: 92.8 pg/mL; IgG: 86.4 pg/mL; dynamic range: 100 pg/mL–10 μg/mL; Validation/time: Human serum; clinical validation with SARS-CoV-2 patient and healthy-control sera; LFIA strip development within approximately 10 min; Raman mapping time 200 s per 40 × 40 μm^2^ area	[108]
Section 4.4; Chemiluminescent smartphone-readable LFA	SARS-CoV-2 nucleoprotein	Recognition/signal element: M13 phage-based chemiluminescent reporter conjugated with antibodies and HRP enzymes; Analytical performance: 25 pg/mL using FluorChem imaging; 100 pg/mL using smartphone reader; Validation/time: Nasal swab extract; clinical nasal swab samples; Nasal swab extraction required at least 2 min; signal readout was performed 6 min after ECL substrate addition using FluorChem or ~3 min after substrate addition using the smartphone reader; complete sample-to-result time was not explicitly summed	[109]
Section 4.4; Immunochromatographic strip	Carcinoembryonic antigen	Recognition/signal element: Phage-display-derived CEA affinity peptide conjugated to AuNPs; Analytical performance: 2.5 ng/mL; Validation/time: Clinical serum samples; 60 min at room temperature for complete color development	[111]

**Table 4:** Representative regulatory pathways and clinical-access status of phage therapy in different countries and regions.

Country/Region	Regulatory or Access Model	Representative Status or Example	Key Translational Implication	Representative Refs.
Belgium	Magistral preparation framework	Belgium has implemented a pragmatic framework centered on magistral preparation of tailor-made phage medicines for individual patients. The Belgian FAMHP also describes magistral preparations as pharmacy-based preparations supported by monographs.	Represents a pharmacy-based individualized preparation model that attempts to reconcile patient-specific phage matching with quality oversight.	[142,143,144]
United States	IND and Expanded Access pathway	FDA allows physicians to submit single-patient Expanded Access requests for investigational drugs, including emergency requests, using Form FDA 3926.	Represents a case-by-case compassionate-use route under FDA oversight rather than routine marketing authorization.	[145,146]
Australia	TGA medicine framework; SAS/AP/clinical-trial pathways	TGA states that human bacteriophage therapies are regulated as medicines. No approved bacteriophage therapy is currently entered in the ARTG, but access may occur through SAS, Authorised Prescriber, or clinical-trial pathways.	Provides a structured special-access model for unapproved bacteriophage therapies while maintaining therapeutic-goods oversight.	[147,148]
France	Compassionate access authorization, currently AAC; historically related to the former ATU framework	ANSM lists anti-*Staphylococcus aureus* phages PP1493 and PP1815 under ongoing AAC status for severe documented bone and osteoarticular infections. ANSM also states that nominative ATUs became AACs after the 2021 reform.	Provides a formal compassionate-access pathway with regulatory monitoring, phagogram-based eligibility, and hospital-restricted use.	[149,150]
Russia	Marketed/off-the-shelf bacteriophage preparations	Microgen’s official product information lists Sextaphage^®^ polyvalent pyobacteriophage as a solution for oral, local, and external administration, with activity against several bacterial groups.	Illustrates the existence of off-the-shelf phage preparations in a medicinal-product context, but this should not be equated with modern RCT-based approval evidence.	[23]
Georgia	Eliava-centered clinical and phage-bank ecosystem	Eliava Institute maintains a large bacterial-strain and phage collection. Eliava Phage Therapy Center provides specialized phage-therapy services in Tbilisi and is part of the Eliava Institute.	Highlights the role of phage banks, strain collections, and dedicated clinical infrastructure in personalized phage therapy.	[151,152]
Poland	Experimental therapy under ethics-approved protocols	The Hirszfeld Institute Phage Therapy Unit provides phage therapy according to a protocol approved by an independent bioethics committee; the treatment is currently conducted under a therapeutic-experiment framework.	Represents an academic, ethics-supervised clinical-access model rather than routine product marketing authorization.	[153]
China/NMPA	General drug clinical-trial and registration framework; no phage-specific public pathway identified	No mature, dedicated, publicly established NMPA pathway for human therapeutic phage products has been clearly implemented. Fixed phage products intended as drugs would need to proceed through general drug or biological-product development pathways, whereas individualized clinical use remains an evolving area.	Represents an emerging but still insufficiently defined regulatory area; cautious description is required until phage-specific NMPA guidance or approved products become publicly available.	[154,155]

## Data Availability

No new data were created or analyzed in this study. Data sharing is not applicable to this article.

## Abbreviations

The following abbreviations are used in this manuscript:

Abbreviation	Full term
AAC	Compassionate access authorization (autorisation d’accès compassionnel)
AI	Artificial intelligence
AMR	Antimicrobial resistance
ANSM	Agence nationale de sécurité du médicament et des produits de santé; French National Agency for Medicines and Health Products
AP	Authorised Prescriber
ARTG	Australian Register of Therapeutic Goods
AST	Antimicrobial susceptibility testing
ATU	Temporary authorization for use (autorisation temporaire d’utilisation)
AuNPs	Gold nanoparticles
BAT	Best available antibiotic therapy
BC/Ppy/RGO	Bacterial cellulose/polypyrrole/reduced graphene oxide
Cas	CRISPR-associated protein
CEA	Carcinoembryonic antigen
CF	Cystic fibrosis
CFGO/CB	Carboxyl-functionalized graphene oxide/carbon black
CFU	Colony-forming unit
CFR	Code of Federal Regulations
COF	Covalent organic framework
CRISPR	Clustered regularly interspaced short palindromic repeats
CVM	Center for Veterinary Medicine
DMC	Data Monitoring Committee
DNA	Deoxyribonucleic acid
dPCR	Digital polymerase chain reaction
DPV	Differential pulse voltammetry
dsDNA	Double-stranded DNA
dsRNA	Double-stranded RNA
ECL	Electrochemiluminescence
EIS	Electrochemical impedance spectroscopy
EPA	Environmental Protection Agency
EU	European Union
FCN	Food Contact Notification
FDA	Food and Drug Administration
FSANZ	Food Standards Australia New Zealand
FSIS	Food Safety and Inspection Service
gfp	Green fluorescent protein
GMP	Good manufacturing practice
GRAS	Generally recognized as safe
GRN	GRAS Notice
HRP	Horseradish peroxidase
ICTV	International Committee on Taxonomy of Viruses
IgG	Immunoglobulin G
IgM	Immunoglobulin M
IND	Investigational New Drug
IV	Intravenous
LFIA	Lateral flow immunoassay
LFA	Lateral flow assay
LOD	Limit of detection
LONO	Letter of No Objection
LPS	Lipopolysaccharide
MALDI-TOF	Matrix-assisted laser desorption/ionization time-of-flight
MDR	Multidrug-resistant
ME	Magnetoelastic
MRSA	Methicillin-resistant *Staphylococcus aureus*
MSSA	Methicillin-susceptible *Staphylococcus aureus*
MWCNT-COOH	Carboxylated multi-walled carbon nanotube
NMPA	National Medical Products Administration
nluc	NanoLuc luciferase
PARIS	Phage anti-restriction-induced system
PAS	Phage–antibiotic synergy
PBS	Phosphate-buffered saline
PCR	Polymerase chain reaction
PDR	Pandrug-resistant
PHEIGES	PHage Engineering by In vitro Gene Expression and Selection
PK/PD	Pharmacokinetics/pharmacodynamics
POCT	Point-of-care testing
qPCR	Quantitative polymerase chain reaction
RCT	Randomized controlled trial
RNA	Ribonucleic acid
RTE	Ready-to-eat
SARS-CoV-2	Severe acute respiratory syndrome coronavirus 2
SAS	Special Access Scheme
SERS	Surface-enhanced Raman scattering
SMART	Splitting, modifying, assembling, and rebooting
SPR	Surface plasmon resonance
ssDNA	Single-stranded DNA
ssRNA	Single-stranded RNA
STAMP	Standardised Treatment and Monitoring Protocol
STEC	Shiga toxin-producing *Escherichia coli*
TGA	Therapeutic Goods Administration
TXTL	Cell-free transcription-translation
UCSD/IPATH	University of California San Diego Center for Innovative Phage Applications and Therapeutics
USDA	United States Department of Agriculture
VAP	Ventilator-associated pneumonia
VRE	Vancomycin-resistant *Enterococcus*
XDR	Extensively drug-resistant
ZIF-8	Zeolitic imidazolate framework-8
